# Investigation of the Pressure Gain Characteristics and Cycle Performance in Gas Turbines Based on Interstage Bleeding Rotating Detonation Combustion

**DOI:** 10.3390/e21030265

**Published:** 2019-03-08

**Authors:** Lei Qi, Zhitao Wang, Ningbo Zhao, Yongqiang Dai, Hongtao Zheng, Qingyang Meng

**Affiliations:** College of Power and Energy Engineering, Harbin Engineering University, Harbin 150001, China

**Keywords:** rotating detonation, gas turbine, pressure gain, entropy change, cycle efficiency, power generation

## Abstract

To further improve the cycle performance of gas turbines, a gas turbine cycle model based on interstage bleeding rotating detonation combustion was established using methane as fuel. Combined with a series of two-dimensional numerical simulations of a rotating detonation combustor (RDC) and calculations of cycle parameters, the pressure gain characteristics and cycle performance were investigated at different compressor pressure ratios in the study. The results showed that pressure gain characteristic of interstage bleeding RDC contributed to an obvious performance improvement in the rotating detonation gas turbine cycle compared with the conventional gas turbine cycle. The decrease of compressor pressure ratio had a positive influence on the performance improvement in the rotating detonation gas turbine cycle. With the decrease of compressor pressure ratio, the pressurization ratio of the RDC increased and finally made the power generation and cycle efficiency enhancement rates display uptrends. Under the calculated conditions, the pressurization ratios of RDC were all higher than 1.77, the decreases of turbine inlet total temperature were all more than 19 K, the power generation enhancements were all beyond 400 kW and the cycle efficiency enhancement rates were all greater than 6.72%.

## 1. Introduction

As one of the most important types of power plants, gas turbines are widely used in the fields of aviation, shipping, power stations, etc. Faced with a greater demand for high efficiency and low pollution, higher level gas turbines have attracted more and more attention in recent years. However, it is quite difficult to improve the cycle efficiency in conventional gas turbines due to the high entropy change during the combustion process [1,2]. In order to break through the bottleneck of efficiency improvement, various advanced combustion technologies, including detonation [3,4,5], wave rotors [6], and shockless explosion [7,8] were studied in the past few decades.

Theoretical pure detonation combustion which has a lower entropy change and self-pressure gain compared with iso-pressure combustion under similar conditions has already attracted wide attention since the beginning of the 21st century [9,10]. Due to the difficulty of the high Mach number environment in the standing oblique detonation combustion process [11] and the high frequency ignition in the pulse detonation combustion process [12], rotating detonation with its wide working range and self-sustaining propagation has been attracting increasing attention. The evolution characteristics of rotating detonation wave [13,14], the thermodynamic characteristics of rotating detonation flow fields [15,16,17,18,19] and the propulsion performance of the rotating detonator combustor (RDC) [20,21,22] have been investigated with a series of experiments and numerical studies.

In recent years, many studies on the application of rotating detonation combustion to gas turbine cycles have been gradually developed. It has been found that rotating detonation combustion could significantly improve the cycle performance of gas turbines. Wolański et al. applied RDC to a GT350 engine and found that stable continuous detonation was difficult to establish for pure Jet-A fuel. The addition of hydrogen fuel could greatly improve the stability of RDC. With the dual fuel including Jet-A and hydrogen, the thermal efficiency showed an increase of 5–7% compared with that of the base engine [23,24]. After a series of studies on the flow field characteristics of a three-dimensional RDC [25,26,27], Frolov et al. explored the feasibility of the application of RDC to gas turbines in 2016. An isolator was designed to dampen the pressure disturbances towards the upstream compressor and a total pressure gain of 15% was found during the combustion course [28]. Naples et al. improved the export structure of the RDC by adding a trailing annular ejector, which reduced the static pressure pulsation at the RDC outlet by 60–70% [29]. On this basis, a new T63 engine with a RDC using hydrogen as fuel was tested in 2017 and the cycle efficiency was improved under some conditions [30]. Combined with the design of a RDC expansion outlet and a supersonic turbine, Sousa analyzed the cycle performance of rotating detonation gas turbines using hydrogen as fuel and found the cycle efficiency could be increased by about 5% at low pressure ratios, but it showed no increase at high pressure ratios [31]. Qi et al. adopted a simplified model to analyze the cycle performance of the rotating detonation gas turbine under different working conditions. The efficiency could be increased by 5–9% compared with the base gas turbine, and equivalence ratio in RDC played an important role in design of the new cycle [32]. In addition, there were also some studies on the connection between RDCs and turbines from an experimental and numerical view, although with no analysis of the engine cycles [33,34,35].

Up to now, plenty of research on gas turbines based on rotating detonation has used a fuel with a small detonation cell size, such as hydrogen and ethylene, which cannot be applied in marine engines and power stations. The little research using industrial fuel either ignored many of the characteristics of rotating detonation flow fluid or brought in extra structures which were difficult to design. In our previous studies, the ejector was simplified as a mixing model including a series of assumptions which will cause many engineering design difficulties. In this paper, a new thermodynamic cycle model of a gas turbine based on interstage bleeding rotating detonation combustion was established using methane as fuel. The air from interstage bleeding participated in combustion and the higher pressure air at the outlet of compressor sprayed into the outlet of RDC combustor for mixing, which will avoid the need for a complex ejector design and makes better use of the air from interstage bleeding. An investigation of the pressure gain characteristics and cycle performance were carried out with a series of numerical simulations and cycle parameter calculations.

## 2. Methodology

### 2.1. Cycle Scheme

**Figure 1:**
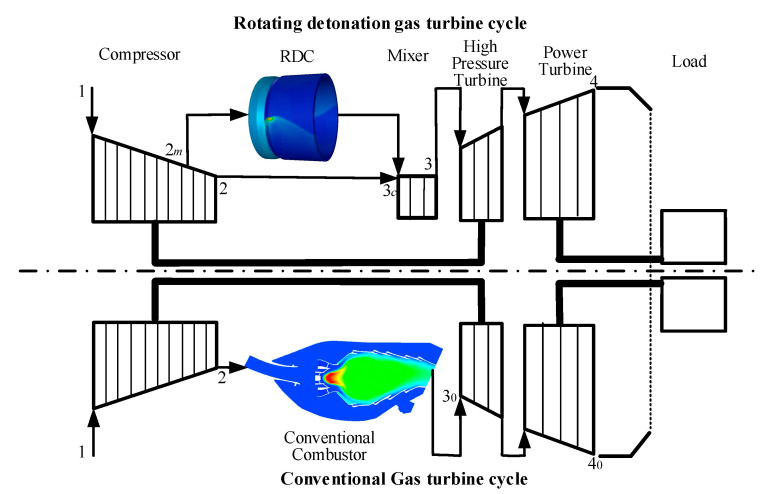
Cycle scheme of a gas turbine with two combustion modes.

**Figure 2:**
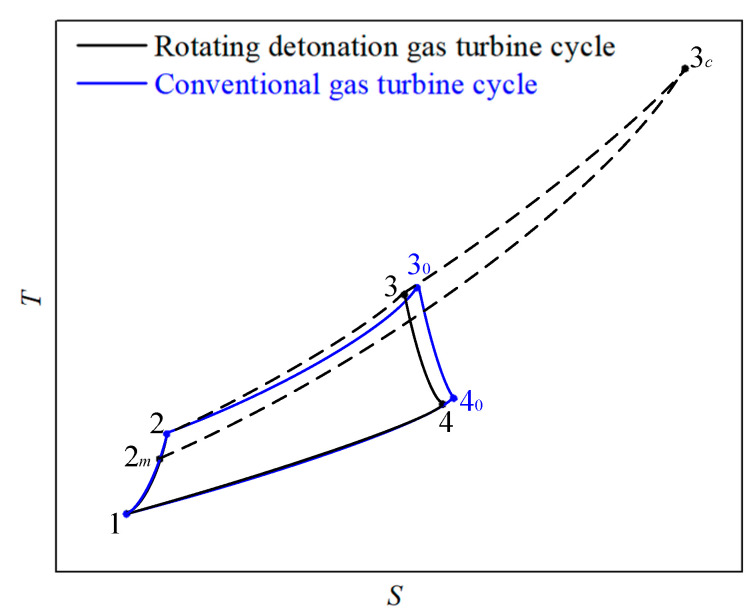
*T-S* diagrams of a marine gas turbine cycle with different combustion processes.

Figure 1 shows the cycle scheme of a gas turbine with two combustion modes. Above is the gas turbine cycle based on interstage bleeding rotating detonation combustion, and below is the conventional gas turbine cycle. Compared with the conventional gas turbine cycle, the bleeding air from a 3/4 compressor entered into the RDC and participates in the pressure gain combustion, the higher pressure air from the compressor outlet enters into a mixer and cools the gas from the RDC outlet. The design complexity of the mixer could be effectively reduced as the air from the compressor outlet had a pressure that is higher or close to that of the air from interstage bleeding. In terms of the cycle scheme, the actual *T-S* diagrams which respectively represent the thermodynamic cycles of the conventional gas turbine and rotating detonation gas turbine are studied in this section, as shown in Figure 2.

### 2.2. Physical Model and Computing Method

In this section, the model information of gas turbine cycle based on interstage bleeding rotating detonation combustion will be mainly described, including the compressor model, combustion model, mixer model, turbine model and other necessary equations. Meanwhile, to visually investigate the performance of the rotating detonation gas turbine cycle, a conventional gas turbine cycle based on approximate iso-pressure combustion (called reference gas turbine cycle, of which power parameters referred to a certain type of gas turbine in practical application) was brought in for reference. The difference of computing method only exists in the combustion progress to analyze the influence of combustion mode on the cycle performance.

- Compressor model (1→2)

As the mass flow rate of air $g_{a}$, compressor inlet temperature $T_{1}^{*}$, compressor inlet pressure $P_{1}^{*}$, compressor efficiency $\eta_{c}$, compressor pressure ratio $\pi_{c}$, compressor temperature ratio τ were known, compressor outlet temperature $T_{2}^{*}$ and compressor outlet pressure $P_{2}^{*}$ of compressor could be obtained by:

(1) $$\eta_{c}=\frac{H_{2s}^{*}-H_{1}^{*}}{H_{2}^{*}-H_{1}^{*}}$$

(2) $$T_{2}^{*}=T_{1}^{*}\tau$$

(3) $$P_{2}^{*}=P_{1}^{*}\pi_{c}$$

where $H_{1}^{*}$ and $H_{2}^{*}$ are the enthalpies at state points of 1 and 2. $H_{2s}^{*}$ is the ideal enthalpy in isentropic condition.

Compressor pressure ratio $\pi_{c}$, compressor outlet temperature $T_{2}^{*}$, compressor outlet pressure $P_{2}^{*}$, interstage bleeding temperature $T_{2m}^{*}$, interstage bleeding pressure $P_{2m}^{*}$, mass flow rate of air $g_{a}$ were calculated at the 100% load (design condition) ~50% load of the reference gas turbine, as shown in Table 1. As the power generations of reference gas turbine $L_{E,0}$ at different loads were known, the state parameters of compressor outlet in different conditions could be obtained through cycle computations.

- Combustion model (2m→3c/2→3_0_)

In this paper, RDC(2m→3c) was simplified to a two-dimensional rectangular model, of which the length and width were respectively 800 mm and 200 mm (these sizes were preliminarily determined by referring to [19,36,37,38]). A more detailed simplified method and its reasonability can be found in [39,40], where the effectiveness of this method has been verified. The density-based solver was chose to solve the two-dimensional unstable Euler Equation. The laminar finite rate model was selected due to the fact viscosity is neglected. In the previous studies such as [16,41,42,43,44], the rotating detonation phenomenon could be effectively described by a laminar finite rate model. In addition, to effectively capture the shock waves and detonation waves, the flux term was dispersed by the advection upstream splitting method (AUSM) [45]. The convective term and time term were, respectively, dispersed in the third-order upwind scheme and using the four-step Runge–Kutta method with the second-order accuracy [46]:

(4) $$\frac{\partial \Phi }{\partial t}+\frac{\partial U}{\partial x}+\frac{\partial V}{\partial y}=\Omega$$

where the dependent variable vector Φ, the convective flux vectors U and V, and the source vector Ω are defined as:

(5) $$\Phi ={\left(\rho ,\rho u,\rho v,\rho e_{t},\rho_{j}\right)}^{T}$$

(6) $$U={\left(\rho u,\rho u^{2}+P,\rho uv,\left(\rho e_{t}+P\right)u,\rho_{j}u\right)}^{T}$$

(7) $$V={\left(\rho v,\rho uv,\rho v^{2}+P,\left(\rho e_{t}+P\right)v,\rho_{j}v\right)}^{T}$$

(8) $$\Omega ={\left(0,0,0,0,\omega_{j}\right)}^{T}$$

where ρ is the density of the premixture, $\rho_{j}$ is the density of species j, u and v are respectively the velocity in *X* and *Y* direction, *P* is the pressure that can be given by the thermal equation of state for a perfect gas. The total internal energy is defined as:

(9) $$e_{t}=e+\frac{1}{2}\left(u^{2}+v^{2}\right)$$

where a caloric equation of state for the internal energy of a reacting mixture:

(10) $$e=e\left(\rho_{j},T\right)$$

**Figure 3:**
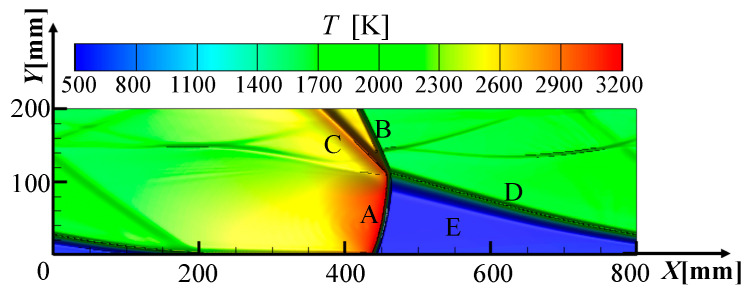
Temperature shadowgraphs of RDC.

Figure 3 shows the temperature shadowgraph of the RDC at *t* = 6800 μs. The temperature characteristics reflected in the temperature contour and extra important pressure characteristic can both be reflected in the temperature shadowgraph.

It could be seen that there were detonation wave A, oblique shock wave B, mixing region C, discontinuity region D and premixed gas region E, which is in good agreement with the published results [47,48,49]. The independence test and model validation have already been carried out in the previous study [19,32], so the grid size and time step were respectively selected as 1 mm and 0.2 μs. In this study, the propagation speed of detonation wave was 1800.4 m/s in the design condition and the theoretical Chapman Jouguet (CJ) propagation velocity of detonation wave is 1808.0 m/s under the same conditions, so the error is only 0.8%. With the parameters calculated in the numerical simulation, the combustion thermal efficiency $\eta_{r}$ pressurization ratio $\pi_{3c}$, the nonuniformities of outlet static pressure *NU_P_* and outlet static temperature *NU_T_* can be obtained using the following equations:

(11) $$\pi_{3c}=P_{3c}^{*}/P_{2m}^{*}$$

(12) $$\eta_{r}=\left(H_{3c}^{*}-H_{2m}^{*}\right)/Q_{in}$$

(13) $$NU_{P}=\sqrt{\sum_{i=1}^{n}{\left(P_{i}-\bar{P}\right)}^{2}/\left(n-1\right)}/\bar{P}$$

(14) $$NU_{T}=\sqrt{\sum_{i=1}^{n}{\left(T_{i}-\bar{T}\right)}^{2}/\left(n-1\right)}/\bar{T}$$

where $P_{3c}^{*}$ and $H_{3c}^{*}$ are the outlet total pressure and outlet total enthalpy at the state point of 3c, $Q_{in}$ represents the heat release of fuel, and $\bar{P}$, $\bar{T}$ are average static pressure and temperature of RDC outlet.

In the conventional approximate iso-pressure combustion progress (2→3_0_) of the reference gas turbine cycle, there was an important parameter—the total pressure recovery coefficient σ—which was applied in the followed equation and defined as 0.934:

(15) $$\sigma =P_{3,0}^{*}/P_{2}^{*}$$

- Mixer model (3c→3)

A mixing process happens in the mixer between the gas of the RDC outlet and the air of the compressor outlet. Two assumptions were made in the mixer model, one was that the all mechanical losses were ignored, the other was that the development of an oblique shock wave stopped in the mixer. Thus, the key parameters in this model can be calculated by:

(16) $$g_{a}\;=\;g_{a,m}+\;g_{a,c}$$

(17) $$\left(g_{a,c}+g_{f}\right)P_{3c}^{*}+g_{a,m}P_{2}^{*}=\left(g_{a}+g_{f}\right)P_{3}^{*}$$

where $g_{a,c}^{}$ is the mass flow rate of the combustion air, $g_{a,m}^{}$ is the mass flow rate of the mixing air, $g_{f}^{}$ is the mass flow rate of the fuel, and $P_{3}^{*}$, $T_{3}^{*}$, $\pi_{3}$ respectively represent the total pressure, total temperature and composite pressure ratio at the state point of 3.

- Turbine model (3→4/3_0_→4_0_)

As the loss coefficient of exhaust pressure $\xi_{out}$ was known, and expansion ratio ($\pi_{t}$) and efficiency ($\eta_{t}$) of turbine were:

(18) $$\pi_{t}=\frac{P_{3}^{*}}{P_{2}^{*}}\pi_{c}\xi_{out}$$

(19) $$\eta_{t}=\frac{H_{3}^{*}-H_{4}^{*}}{H_{3}^{*}-H_{4s}^{*}}$$

where $H_{3}^{*}$ and $H_{4}^{*}$ are the enthalpies at state points of 3 and 4. $H_{4s}^{*}$ is the ideal enthalpy in isentropic condition (the equations were the same in the 3_0_→4_0_ case)

Based on the above models and the necessary iterative computations, the following thermodynamic cycle performance parameters can be obtained:

Compressor input power $L_{C}$:

(20) $$L_{C}=g_{a,c}\int_{T_{1}^{*}}^{T_{2}^{*}}C_{P}(T)dT\;+\;g_{a}\int_{T_{1}^{*}}^{T_{2m}^{*}}C_{P}(T)dT$$

Turbine output power $L_{T}$:

(21) $$L_{T}=\left(g_{a}+g_{f}\right)\int_{T_{4}^{*}}^{T_{3}^{*}}C_{P}(T)dT$$

Power generation $L_{E}$:

(22) $$L_{E}=L_{T}\;-\;L_{C}$$

Cycle efficiency η:

(23) $$\eta =L_{E}/Q_{in}$$

Cycle efficiency increasing rate $w_{\Delta \eta }$:

(24) $$w_{\Delta \eta }=\frac{\eta -\eta_{0}}{\eta_{0}}\times 100\%$$

In addition, as the fuel of the gas turbine can be completely combusted, the gas composition mainly included N_2_, O_2,_ CO_2_, and H_2_O. In view of the additivity of $C_{P}$ for the mixed gas, the following equation was used:

(25) $$C_{P}(T)=8.3145(A_{1}+A_{2}T+A_{3}T^{2}+A_{4}T^{3}+A_{5}T^{4})$$

where *A* is coefficient related to composition, obtained in [50].

The enthalpy of each point was calculated by:

(26) $$H=\int_{T_{ref}^{}}^{T}C_{P}(T)dT$$

Entropy of any point S:

(27) $$S_{}=\int_{T_{\mathrm{ref}}^{}}^{T}\frac{C_{P}(T)}{T}dT-R_{g}\ln\frac{P}{P_{ref}}$$

where $T_{ref}^{}$ is the reference temperature, 288.15 K, and $P_{ref}^{}$ is the reference pressure, 0.1013 MPa.

The efficiency characteristics of the compressor and turbine were referenced from universal characteristic lines, obtained in [51,52].

## 3. Results and Discussion

### 3.1. Flow Field Characteristic Research of RDC

**Figure 4:**
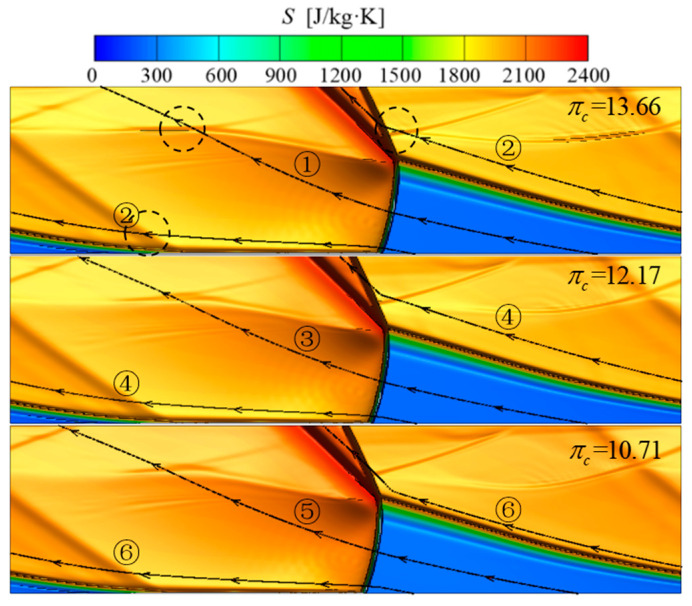
Entropy shadowgraphs of the RDC at 6800 μs.

Figure 4 presents entropy shadowgraphs at *t* = 6800 μs. In the axial direction, the height of the detonation wave was about 95 mm, the height of the oblique shock wave was about 90 mm, and the height of the transition region was about 15 mm at different compressor pressure ratios. The six path lines passed through the midpoint of detonation wave (type 1 of path lines) and oblique shock wave (type 2 of path lines) in the axial direction, respectively. The path lines were obtained by transformation of coordinates, which was explained in details in [19,41]. Path lines ①, ③, ⑤ passed through detonation wave at about *Y* = 50 mm and a weak shock wave at about *Y* = 150 mm. Path lines ②, ④, ⑥ passed through the detonation wave at about *Y* = 7.8 mm, weak shock waves at about *Y* = 25 mm, 150 mm and the oblique shock wave at about *Y* = 155 mm.

**Figure 5:**
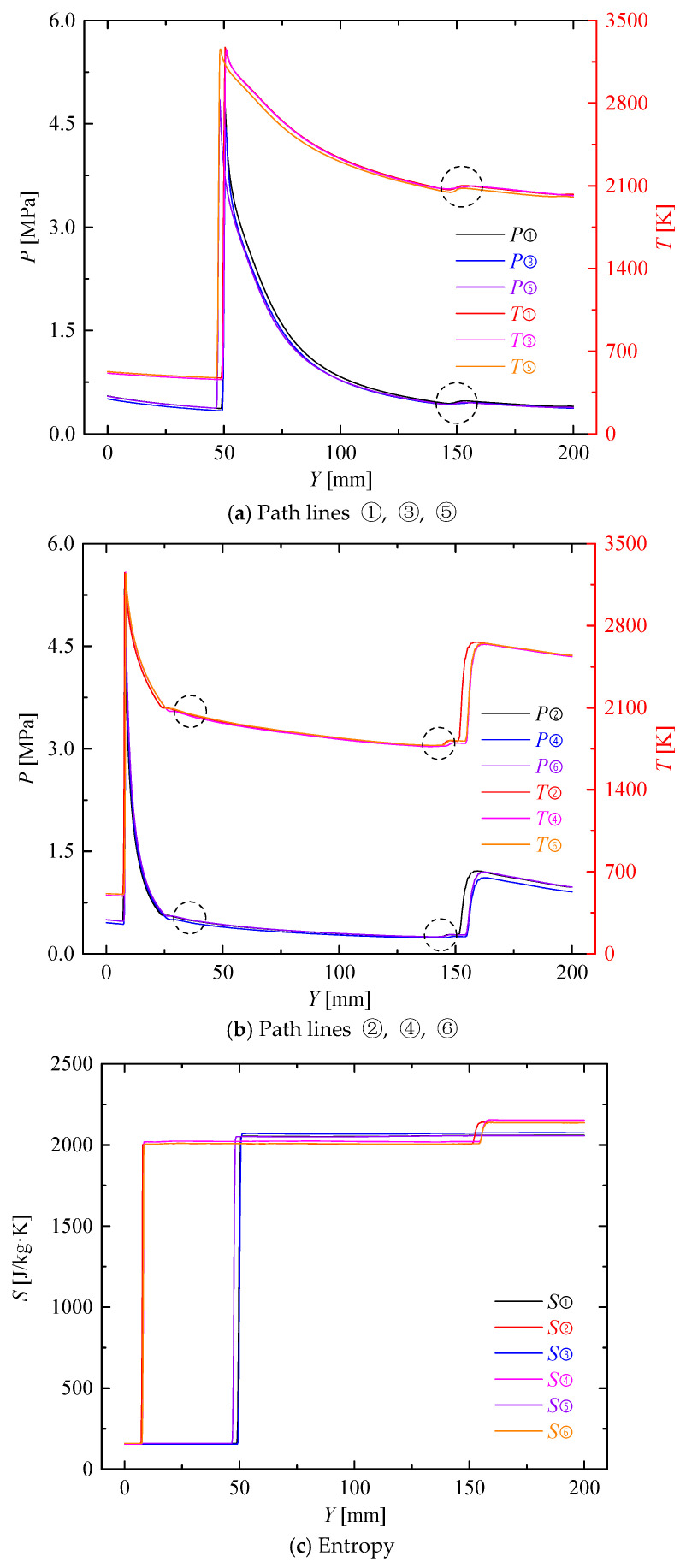
Variations of pressure, temperature and entropy along different path lines.

The changes of static temperature and static pressure along path lines ①, ③, ⑤ are shown in Figure 5a. Path lines passed through the detonation wave at about *Y* = 47.5 mm, where the pressure and temperature both displayed jumps and then gradually decreased. At *Y* = 150 mm, the pressure and temperature shows a smaller jump due to the fact the path lines are passing through a weak shock wave, where the pressure increased by 8.07%, 5.22%, 8.27% and the temperature increased by 1.66%, 1.16%, 1.75%. In all, the pressure decreased by 27.34%, 26.96% 30.44% from the beginning to the end, respectively.

Figure 5b presents the changes of static temperature and static pressure along path lines ②, ④, ⑥. Pressure and temperature had the first big jumps when the path lines passed through the detonation wave at about Y = 7.8 mm. Then the two parameters dropped extremely. Path lines ②, ④, ⑥ passed through the first weak shock wave at *Y* = 25 mm. Although the two parameters had no significant change, the slope of downtrend slowed down obviously. Path lines ②, ④, ⑥ passed through the second weak shock wave at *Y* = 150 mm, where the two parameters decreased less than 1%. Pressure and temperature had their second big jumps when the path lines passed through the oblique shock wave at *Y* = 155 mm. In all, the pressure increased by 96.05%, 99.45%, 96.30% from the beginning to the end, respectively.

Figure 5c shows the change of the entropy along the six path lines. The same as the change trend of temperature, big jumps happened when the path lines passed through the detonation wave. At the moment, the entropy change reached about 1900 J/kg·K along path lines ①, ③, ⑤ and about 1850 J/kg·K along path lines ②, ④, ⑥. Another jump happened when the path lines ②, ④, ⑥ passed through the oblique shock wave. As for path lines ②, ④, ⑥, the major entropy change was rooted in the detonation region and the second entropy change led by the oblique shock wave was about 6.61% of the total. In all, the entropy change reached about 1900 J/kg·K and 2000 J/kg·K for the two styles of path lines from the beginning to the end, respectively. In addition, although the weak shocks caused sudden changes in the pressure and temperature diagram, it had no significant effect on the entropy change.

**Figure 6:**
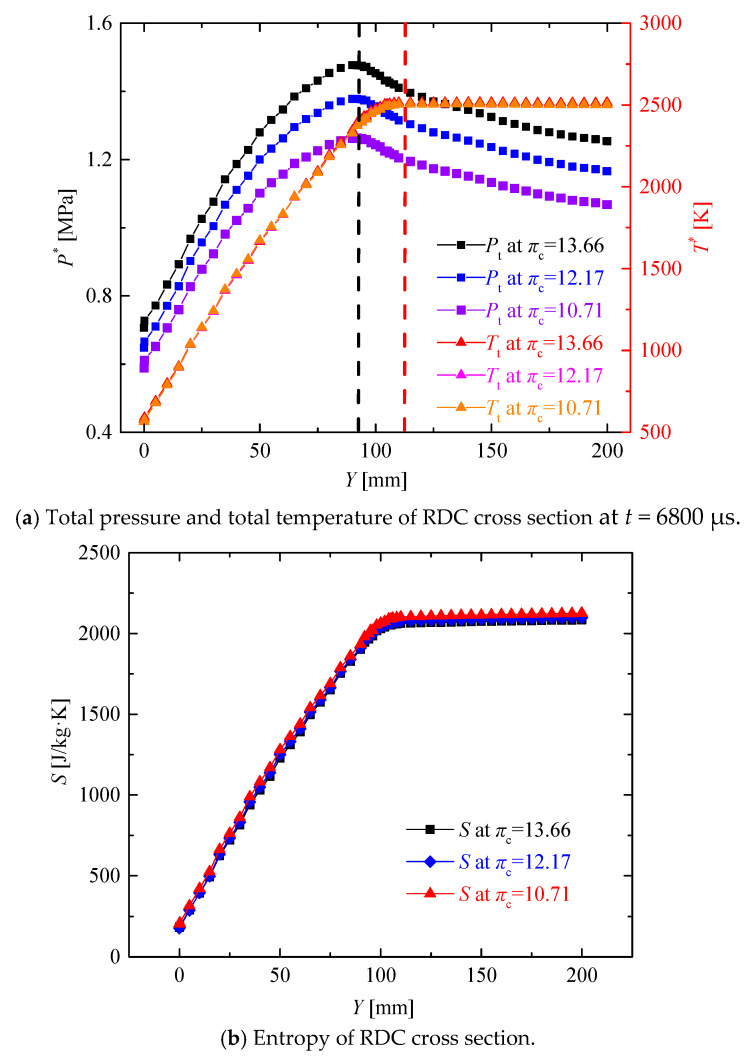
Mass-weighted average parameters of the RDC cross-section.

As there was a significant difference in the pressure changes along the two types of path lines, mass-weighted average parameters of the RDC cross-section in the axial direction were analyzed, as shown in Figure 6a. Combined with the heights of the detonation wave, oblique shock wave and transtion region as mentioned above, the total pressure $P^{*}$ and total temperature $T^{*}$ increased significantly due to the existence of the detonation wave, and the total pressure reached the highest value at the end point of the detonation wave at *Y* = 95 mm. Entering into the transtion region including deflagration combustion, the total pressure started to decrease and the total temperature reached its peak at the end of transtion region when the combustion was completed. In the region including the oblique shock wave, the total pressure continued to decrease but the total temperature remain unchanged. In general, there were significant pressurization characteristics in the RDC, of which the instantaneous pressurization ratios respectively reached 1.7757, 1.8005, and 1.8150 at different compressor ratios. Figure 6b presents the entropy of the RDC cross-section in the axial direction. The entropy kept increasing from the inlet to the outlet of RDC. The slope was the largest in the detonation combustion region, decreased in the transition region and reached a minimum in the oblique shock wave region.

### 3.2. Variations of RDC Characteristic Parameters at Different Compressor Pressure Ratios

The mass flow rate of air was divided into two parts: the mass flow rate of bleeding air from the 3/4 compressor which will enter into the RDC was defined as $g_{a,c}$, and the mass flow rate of the higher pressure air from the compressor outlet was defined as $g_{a,m}$. Table 2 presents the mass flow rates and distribution proportions of the two parts of air when the equivalent ratio of the RDC is 1.

**Figure 7:**
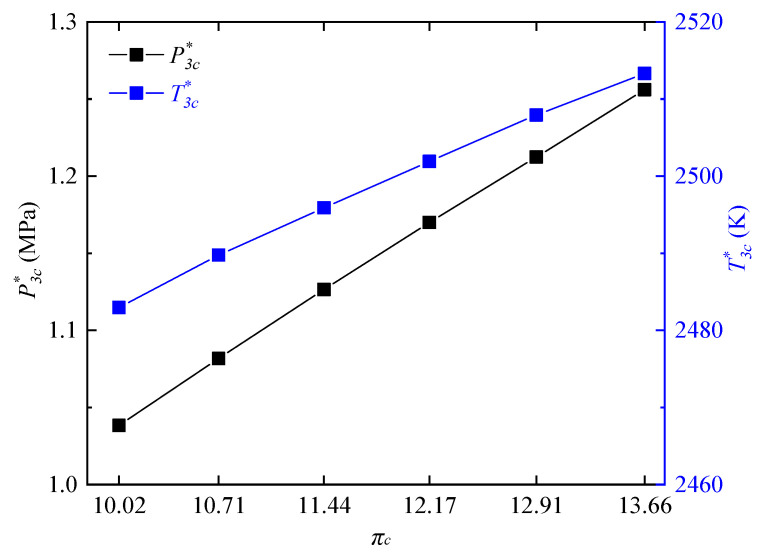
Variations of $T_{3c}^{*}$ and $P_{3c}^{*}$ at different compressor pressure ratios.

Figure 7 shows the variations of outlet total pressure $P_{3c}^{*}$ and outlet total temperature $T_{3c}^{*}$ of RDC at different compressor pressure ratios. All the following data of the RDC outlet were calculated using the mass-weighted and time average method [32]. The two parameters both had a downtrend with the decrease of compressor pressure ratio. When the compressor pressure ratio decreased from 13.66 to 10.02, $P_{3c}^{*}$ and $T_{3c}^{*}$ decreased from 1.2561 MPa, 2513.3 K to 1.0383 MPa, 2482.95 K, respectively.

**Figure 8:**
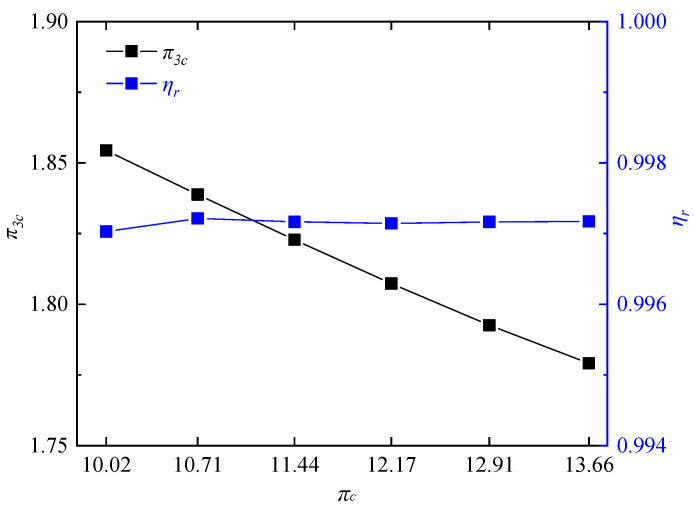
Variations of $\pi_{3c}^{}$ and $\eta_{r}^{}$ at different compressor pressure ratios.

**Figure 9:**
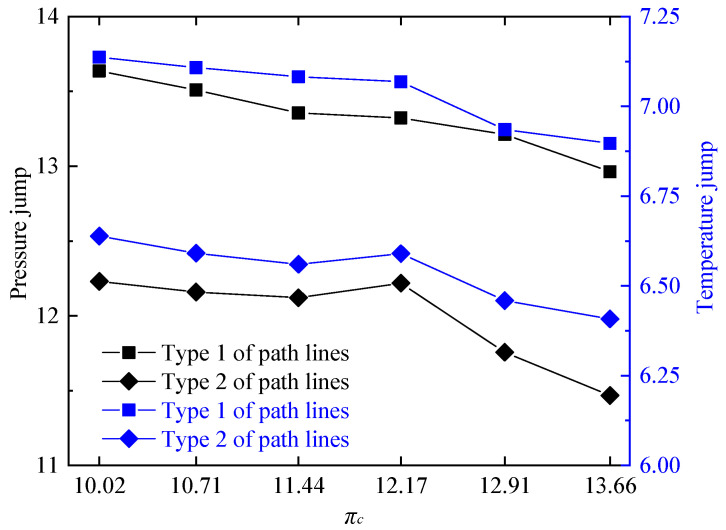
Variations of temperature and pressure jumps at different compressor pressure ratios.

In order to further investigate the performance of RDC, the pressurization ratio and combustion thermal efficiency of the RDC were calculated under different working conditions according to the Equations (11) and (12), as shown in Figure 8. It can be seen that as the compressor pressure ratio decreased, $\eta_{r}$ of the RDC did not change significantly, all being beyond 0.9971. Additionally, $\pi_{3c}$ gradually increased with the decrease of compressor pressure ratio. At $\pi_{c}$ = 13.66, $\pi_{3c}$ reached 1.7791. When $\pi_{c}$ decreased to 10.02, $\pi_{3c}$ increased by 4.3%, reaching 1.8545. From the view of variation of flow field parameters, higher temperature jumps and pressure jumps in the detonation wave would promote a higher pressure gain in the RDC [53]. Therefore, variations of temperature and pressure jumps were calculated along each of the two types of path lines at different compressor pressure ratios, as shown in Figure 9. Choices of the typical path lines were same as those in Section 3.1, respectively, passing through the midpoint of the detonation wave (type 1 of path lines in Figure 9) and the midpoint of the oblique shock wave (type 2 of path lines in Figure 9). With the decrease of compressor pressure ratio, pressure and temperature jumps at the detonation wave both had an uptrend, which was in accordance with the variation trend seen in Figure 8.

**Figure 10:**
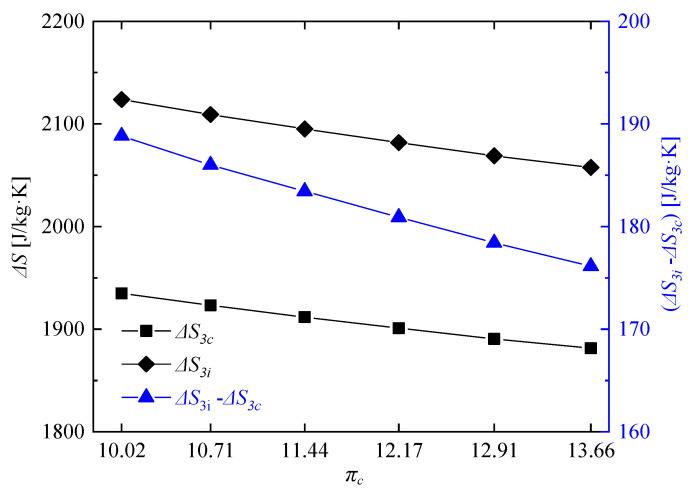
Variations of related parameters of the entropy change at different compressor pressure ratios (Δ*S*_3*c*_ = *S*_3*i*_ − *S*_3*c*_).

Furthermore, the entropy change was analyzed to explain the reason for the variation of pressure gain characteristics. As the pressure and temperature both had obvious influences on the entropy change, the entropy change of iso-pressure combustion at the same compressor pressure ratio was used for reference to eliminate the influence of temperature, so the entropy change will only be reflected in the change of pressure. Figure 10 shows the related parameters of the entropy change. As the compressor pressure ratio decreased, both the entropy change of RDC *S*_3*c*_ and the entropy change of iso-pressure combustion *S*_3*i*_ increased. The difference between them Δ*S*_3*c*_ (Δ*S*_3*c*_ = *S*_3*i*_ − *S*_3*c*_) also had the same uptrend, which meant that the available energy loss savings in the rotating detonation combustion gradually increased with the decrease of compressor pressure ratio, and was finally reflected in the higher and higher total pressure of the RDC outlet. The change of entropy change increased from 176.14 J/kg·K to 188.83 J/kg·K when the compressor pressure ratio decreased from 13.66 to 10.02.

**Figure 11:**
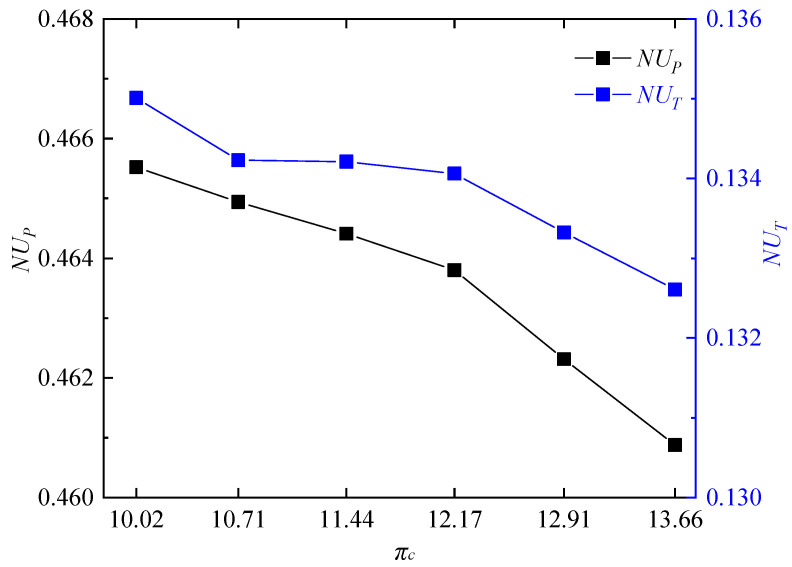
Non-uniformities of the outlet static pressure and outlet static temperature.

In addition, the non-uniformities of the outlet static pressure and outlet static temperature were calculated at different compressor pressure ratios. As shown in Figure 11, the nonuniformities of outlet static pressure *NU_P_* and outlet static temperature *NU_T_* both had uptrends with the decrease of compressor pressure ratio, but with a small variation range. However, the non-uniformity of outlet static pressure has already reached to 0.46, which sets a quite high demand for the design of the mixer and first stage blade of the turbine.

### 3.3. Investigation of Cycle Performance in Gas Turbine Based on Interstage Bleeding Rotating Detonation Combustion

**Figure 12:**
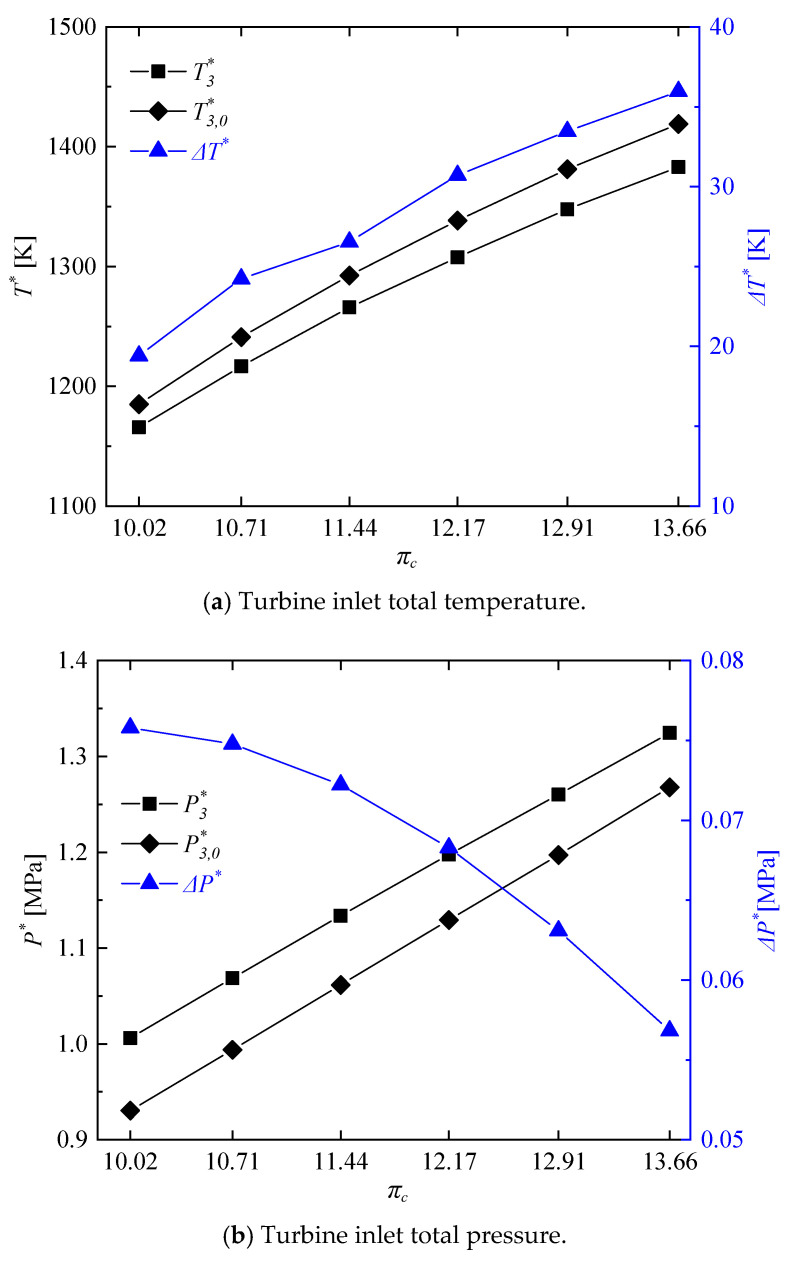
Variations of turbine inlet total temperature and turbine inlet total pressure at different compressor pressure ratios.

Figure 12 shows the parameters related to the turbine inlet total temperature and turbine inlet total pressure of the rotating detonation gas turbine and reference gas turbine at different pressure ratios.

**Figure 13:**
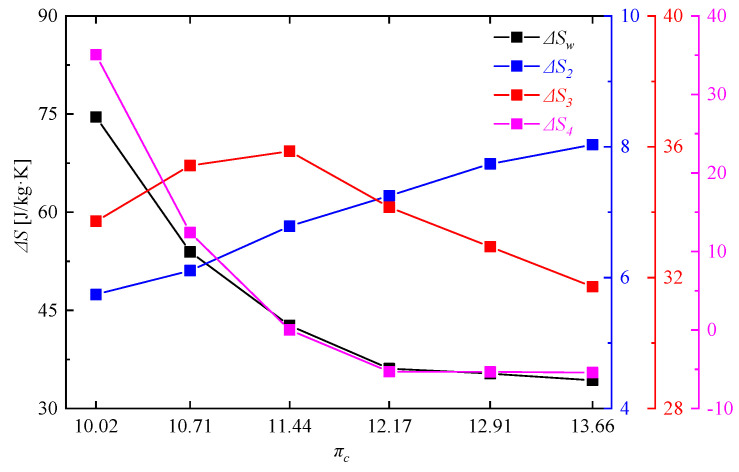
Difference of entropy change between the reference gas turbine and the rotating detonation gas turbine.

It can be seen in Figure 12a that as the compressor pressure ratio decreased from 13.66 to 10.22, the turbine inlet total temperature of the rotating detonation gas turbine decreased from 1382.90 K to 1165.69 K. Moreover, there was a decreasing difference compared with those of the reference gas turbine, but all beyond 19 K. As shown in Figure 12b, with the decrease of compressor pressure ratio, the difference of turbine inlet total pressure between the reference gas turbine and rotating detonation gas turbine gradually increased, but with a decreasing slope. The reason for this phenomenon was that when the compressor pressure ratio was too low, the pressurization ratio of the RDC would be greater than the pressure ratio of the last 1/4 compressor. At that moment, the pressure of air from the compressor outlet is less than the pressure of gas from the RDC outlet, which could have a negative effect on the pressure in the mixer. The occurrence of this phenomenon led to the uncertainty about the change of cycle efficiency, which will be analyzed in details in the last part of this paper. Figure 13 shows the difference of entropy change between the reference gas turbine and the rotating detonation gas turbine, including the differences in the whole working progress Δ*S_W_*, compressor Δ*S*_2_, combustor with mixer Δ*S*_3_, and turbine Δ*S*_4_. With the decrease of compressor pressure ratio, Δ*S*_2_ gradually decreased, Δ*S*_4_ increased, Δ*S*_3_ first increased and then decreased with a turning point at $\pi_{c}$ = 11.44. The turbine played a decisive role in the entropy change of whole working progress, as the uptrend of Δ*S_W_* was similar to Δ*S*_4_. Although a turning point appeared in the variation trend of the combustor with mixer, there was no indication that the slope of Δ*S_W_* was slowing down.

**Figure 14:**
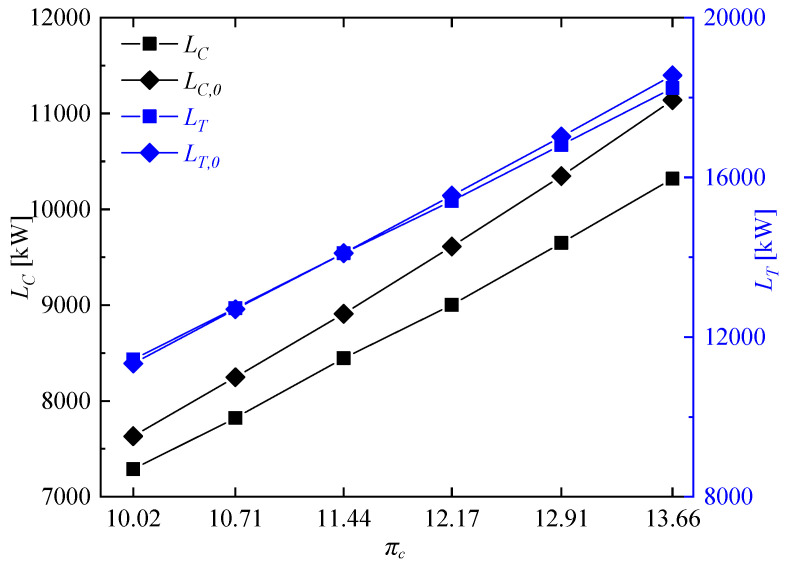
Variations of compressor input power and turbine output power at different compressor pressure ratios.

**Figure 15:**
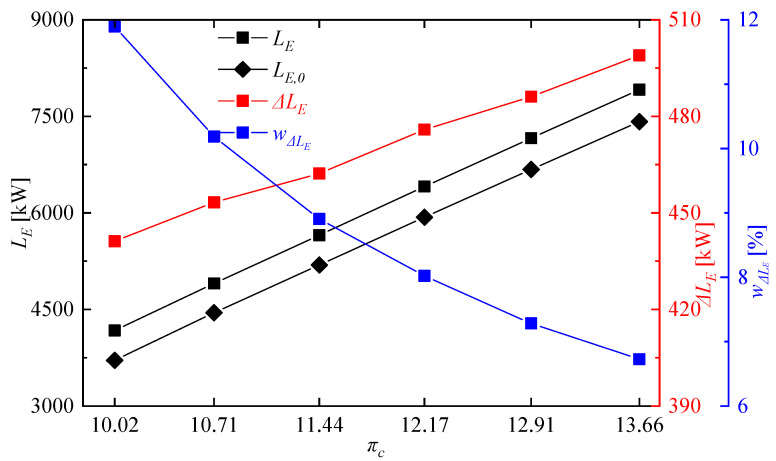
Variation of power generation at different compressor pressure ratios.

**Figure 16:**
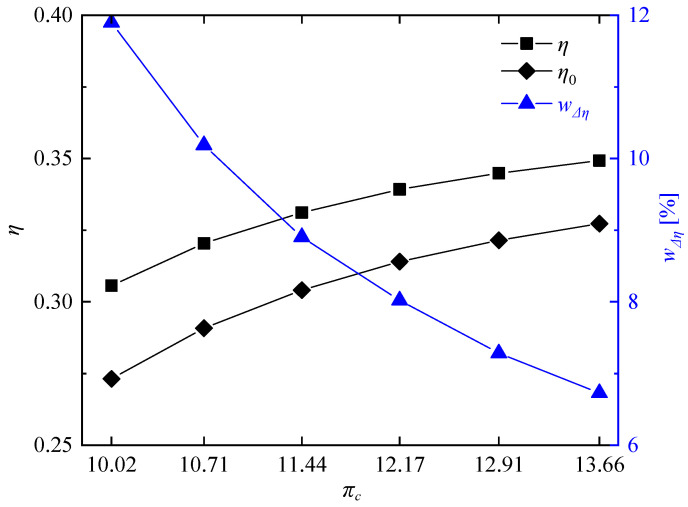
Variation of cycle efficiency at different compressor pressure ratios.

Figure 14 shows compressor input power and turbine output power of the rotating detonation gas turbine and the reference gas turbine at different compressor pressure ratios. It can be clearly seen there is no significant difference of turbine output power between the two gas turbines, but the compressor input power of the rotating detonation gas turbine was less than that of the reference gas turbine, especially at high compressor pressure ratio. Figure 15 presents the variation of power generation at different compressor pressure ratios. With the decrease of compressor pressure ratio, the difference Δ*L_E_* between the power generation of the rotating detonation gas turbine *L_E_* and the power generation of the reference gas turbine *L_E,0_* gradually decreased, but the power generation enhancement rate $w_{\Delta L_{E}}$ had the opposite trend. Δ*L_E_* reached 498.95 kW at $\pi_{c}$ = 13.66, and the power generation of the rotating detonation gas turbine reached 7915.20 kW at that moment. When the compressor pressure ratio decreased to 10.02, a difference of power generation of about 441.18 kW still can be obtained. Meanwhile, the power generation enhancement rate increased from 6.72% to 11.89%. Figure 16 shows the related parameters of cycle efficiency at different compressor pressure ratios. At $\pi_{c}$ = 13.66, the cycle efficiency of the rotating detonation gas turbine η and reference gas turbine $\eta_{0}$ were respectively 0.3493 and 0.3273, with an efficiency enhancement rate ($w_{\Delta \eta }$) of about 6.72%. The two cycle efficiencies decreased to 0.3073 and 0.2731 at $\pi_{c}$ = 10.02, but with an increasing efficiency enhancement rate of about 11.89%. In all the calculated cases, the efficiency enhancement rates of the rotating detonation gas turbine were all greater than 6.72% and increased with the decrease of compressor pressure ratio. In addition, the slope of the efficiency increase rate was approximately stable and had no downward trend with the decrease of compressor pressure ratio. In the previous discussion, the air pressure from the compressor outlet was less than the pressure of gas from the RDC outlet when the compressor pressure ratio was too low, which could have a negative effect on the pressure in the mixer. However, the changes of efficiency enhancement rate showed that the high pressure gain characteristic at low compressor pressure ratio still played a leading role in the variation of cycle efficiency.

The new style gas turbine cycle based on interstage bleeding rotating detonation combustion finally brought an increase about 0.03 in the cycle efficiency of the gas turbine. There was still a gap compared with that described in [32], but this does not mean the research was meaningless. Firstly, from the viewpoint of structural design, enormous convenience was brought in the design of mixer as the pressure of the mixing air (pressurizing in the last 1/4 compressor) was greater than or close to the RDC outlet pressure.

Moreover, the air of interstage bleeding for anti-surge could be better utilized, reducing the loss in the cycle. In summary, it was believed that interstage bleeding rotating detonation combustion could play an important role as an auxiliary combustion system in the gas turbine cycle. Therefore, a gas turbine cycle with combined combustion type (including conventional approximate iso-pressure combustion and rotating detonation combustion) should be further investigated in the future.

## 4. Conclusions

In this paper, a gas turbine cycle model based on interstage bleeding rotating detonation combustion was established using methane as fuel. Combined with a series of two-dimensional numerical simulations and calculations of cycle parameters, variations of pressure gain characteristic and cycle performance were investigated at different compressor pressure ratios. The main conclusions are as follows:

(1) There were significant differences of pressure variation along different types of path lines, but the pressurization characteristics of the RDC were obvious in general.
(2) Compared with the conventional gas turbine cycle, the non-negligible pressure gain characteristics acquired in the interstage bleeding rotating detonation combustor contributed to an obvious performance improvement in the rotating detonation gas turbine cycle. The power generation enhancements were all beyond 400 kW and the cycle efficiency enhancement rates were all greater than 6.72%. Additionally, the turbine inlet total temperature showed a decrease of over 19 K.
(3) Due to the differences of entropy change between iso-pressure combustion and rotating detonation combustion, the pressurization ratio of the RDC increased with the decrease of compressor ratio, which would have a leading influence on the performance improvement in the rotating detonation gas turbine cycle. When the compressor pressure ratio decreased from 13.66 to 10.02, the pressurization ratio of the RDC increased from 1.7791 to 1.8545, then the power generation enhancement rate and cycle efficiency enhancement rate increased from 6.72% to 11.89%.
(4) Compared with the reference gas turbine cycle, the difference of turbine entropy change in the rotating detonation gas turbine cycle played a decisive role in the entropy change of whole working process.

## Figures and Tables

**Table 1:** Relative parameters of the compressor.

**Load**	100%	90%	80%	70%	60%	50%
$L_{E,0}$ **[** **kW** **]**	7416.25	6674.63	5933.00	5191.38	4449.75	3708.13
$\pi_{c}$	13.66	12.90	12.17	11.44	10.71	10.02
$P_{2}^{*}$ **[Mpa]**	1.3573	1.2818	1.2091	1.1365	1.0641	0.9962
$T_{2}^{*}$ **[K]**	688.32	677.44	665.22	652.89	639.65	627.18
$P_{2m}^{*}$ **[Mpa]**	0.7060	0.6763	0.6474	0.6180	0.5882	0.5598
$T_{2m}^{*}$ **[K]**	562.64	555.61	547.71	539.71	531.39	522.96
$g_{a}$ **[** **kg** **/** **s** **]**	26.8185	25.6773	24.6146	23.6218	22.6886	21.8212

**Table 2:** Related parameters of the mass flow rate at different compressor pressure ratios.

$\pi_{c}$	13.66	12.90	12.17	11.44	10.71	10.03
$g_{f}$ **[** **kg** **/** **s** **]**	0.4863	0.4456	0.4055	0.3664	0.3284	0.2914
$g_{a,c}$ **[** **kg** **/** **s** **]**	8.3483	7.6492	6.9598	6.2897	5.6371	5.0013
$g_{a,m}$ **[** **kg** **/** **s** **]**	18.4702	18.0281	17.6549	17.3321	17.0515	16.8199
$g_{a,m}/g_{a,c}$	2.2124	2.2368	2.5367	2.7556	3.0249	3.3631
